# Birth Weight and Long-Term Overweight Risk: Systematic Review and a Meta-Analysis Including 643,902 Persons from 66 Studies and 26 Countries Globally

**DOI:** 10.1371/journal.pone.0047776

**Published:** 2012-10-17

**Authors:** Karen Schellong, Sandra Schulz, Thomas Harder, Andreas Plagemann

**Affiliations:** Clinic of Obstetrics, Division of ‘Experimental Obstetrics’, Charité – University Medicine Berlin, Campus Virchow-Klinikum, Berlin, Germany; Universidad Peruana de Ciencias Aplicadas (UPC), Peru

## Abstract

**Background:**

Overweight is among the major challenging health risk factors. It has been claimed that birth weight, being a critical indicator of prenatal developmental conditions, is related to long-term overweight risk. In order to check this important assumption of developmental and preventive medicine, we performed a systematic review and comprehensive meta-analysis.

**Methods and Findings:**

Relevant studies published up to January 2011 that investigated the relation between birth weight and later risk of overweight were identified through literature searches using MEDLINE and EMBASE. For meta-analysis, 66 studies from 26 countries and five continents were identified to be eligible, including 643,902 persons aged 1 to 75 years. We constructed random-effects and fixed-effects models, performed subgroup-analyses, influence-analyses, assessed heterogeneity and publication bias, performed meta-regression analysis as well as analysis of confounder adjusted data. Meta-regression revealed a linear positive relationship between birth weight and later overweight risk (*p*<0.001). Low birth weight (<2,500 g) was found to be followed by a decreased risk of overweight (odds ratio (OR) = 0.67; 95% confidence interval (CI) 0.59–0.76). High birth weight (>4,000 g) was associated with increased risk of overweight (OR = 1.66; 95% CI 1.55–1.77). Results did not change significantly by using normal birth weight (2,500–4,000 g) as reference category (OR = 0.73, 95% CI 0.63–0.84, and OR = 1.60, 95% CI 1.45–1.77, respectively). Subgroup- and influence-analyses revealed no indication for bias/confounding. Adjusted estimates indicate a doubling of long-term overweight risk in high as compared to normal birth weight subjects (OR = 1.96, 95% CI 1.43–2.67).

**Conclusions:**

Findings demonstrate that low birth weight is followed by a decreased long-term risk of overweight, while high birth weight predisposes for later overweight. Preventing *in-utero* overnutrition, *e.g.*, by avoiding maternal overnutrition, overweight and/or diabetes during pregnancy, might therefore be a promising strategy of genuine overweight prevention, globally.

## Introduction

Overweight is among the top challenging health problems at the beginning of the 21^st^ century [1]. Prevalence has increased alarmingly, reaching epidemic levels in adults, adolescents, and even children in the US and globally [1]–[3]. Across the age spectrum, critical metabolic and cardiovascular morbidity (type 2 diabetes, hypertension, metabolic syndrome, coronary heart disease, stroke) is causally linked to obesity [2], [4], [5]. Cardiovascular and all-cause mortality are strongly related to overweight and obesity irrespective of age, sex, and ethnicity [6]–[9]. Therefore, measures of primary, genuine prevention are urgently needed.

For some years, the ‘fetal origins hypothesis’ on early causes of later diseases has become one of the most promising theoretical frameworks in medicine [10]–[12]. Especially, birth weight has been suggested and used as basic indicator to establish these highly influential concepts [10], [12], since it is decisively determined by the prenatal developmental conditions [13], [14]. However, one of the most important axioms in this context has not been tested globally so far. Although overweight is of central pathogenetic importance for metabolic, cardiovascular and general morbidity and mortality [2], [4], [5], no comprehensive analysis has investigated whether the risk of becoming overweight is related to birth weight in children, adolescents, and adults, *i.e.*, for the long-term.

Therefore, we aimed to characterize this overall critical aspect of the ‘fetal origins’ approach by a systematic review and meta-analysis, substantially extending previous preliminary reviews [15]–[17].

## Methods

### Search strategy and selection criteria

Systematic review and meta-analysis were conducted according to the PRISMA statement for meta-analysis of observational studies (Text S1) [18], including the preparation of a protocol and analysis plan (Text S2). We performed a comprehensive literature search, including the databases MEDLINE and EMBASE (1966–January 2011), to identify studies that investigated the relation between birth weight and later risk of overweight. Searched terms were “birth weight”, “overweight”, “obesity” and “adiposity”, without language restrictions. Furthermore, we manually searched all references cited in original studies and all reviews identified. Authors were contacted if data, methods and/or parameter definitions provided from the respective studies remained unclear.

To be eligible for meta-analysis, a study had to fulfill the following criteria, defined *a priori*: 1) It had to be an original report on the relation between birth weight and risk of overweight. 2) Odds ratios (OR) and 95% confidence intervals (95% CI) (or data with which to calculate them) for risk of overweight in at least two strata of birth weight had to have been reported. All studies which reported the proportion of overweight or obese subjects in at least one age at follow up were included. We did not restrict to a particular definition of overweight/obesity as studies may have been published before currently accepted definitions were introduced [16]. The majority of studies used body mass index (BMI) as overweight criterion in childhood, adolescence as well as adulthood (80%).

From all eligible studies, data were abstracted in duplicate, using a standardized form. An independent reviewer confirmed all data entries.

### Statistical analysis

#### Dichotomous comparisons

We extracted data on numbers of subjects with and without overweight above or below the cutoff value and calculated corresponding crude odds ratios and 95% confidence intervals. We constructed fixed-effects as well as random-effects models to estimate the pooled odds ratios for risk of overweight above *vs.* below the respective cutoff value across all studies.

#### Assessment of heterogeneity

By calculating the I^2^ according to Higgins et al [19], we assessed heterogeneity. Ranging from 0 to 100%, I^2^ is a direct measure of inconsistency of study results in a meta-analysis, with 0% indicating no inconsistency.

#### Influence analysis

Robustness of the pooled estimates was checked by influence analyses. Each of the studies was individually omitted from the data set, followed in each case by recalculation of the pooled estimate of the remaining studies.

#### Subgroup/Sensitivity analyses

To identify potential sources of heterogeneity and sources of bias, studies were stratified by study design and source of birth weight data to assess potential recall bias. Additionally, studies were stratified by publication language. To examine participation/selection bias, we stratified by extent of lost to follow-up. Further stratifications were made by geographic origin, age, overweight classification criterion, source of overweight data, gender distribution, gestational age and parental overweight (BMI>25 kg/m^2^). To assess the impact of parental socioeconomic status (SES), we stratified by the extent by which low SES was present in the study samples.

#### Publication bias

Publication bias was assessed by inspection of the funnel plot and formal testing for funnel plot asymmetry, using Begg's test and Egger's test.

#### Meta-regression

To explore the shape of the continuous relation between birth weight and later overweight risk, meta-regression technique was applied [20]. Accordingly, birth weight- specific odds ratios were related to the respective birth weight. Since birth weight was reported as categorical data with a certain range in the studies (per example, 2,000–2,500 g, 3,000–3,500 g etc.), median of the upper and lower limits of each category was assigned to the particular estimate in each study [21]. Estimates were plotted against respective birth weight as independent variable. After visual inspection, we primarily decided to use a linear regression model. Additionally, fractional polynomial regression was applied because it does not make an *a priori* assumption on shape of the curve. The family of second-order fractional polynomial models provides rich and flexible shapes of curves by choosing p = (p_1_,…,p_m_) as real-valued vector of fractional power from a predefined set [22]. All estimates were weighted by 1/variance.

#### Analysis of confounder-adjusted data

To perform meta-analysis of confounder-adjusted data we considered all studies which reported adjusted odds ratios for risk of overweight for the birth weight categories <2,500 g and/or >4,000 g. Resulting pooled odds ratios were based, however, on different reference categories, as defined by the authors themselves, and therefore not directly comparable to the pooled unadjusted odds ratios evaluated for <2,500 g *vs.* >2,500 g and >4,000 g *vs.* <4,000 g, respectively. To make pooled adjusted and unadjusted odds ratios more comparable, we therefore additionally calculated in all studies which provided adjusted data an unadjusted odds ratio that considered the reference category as used self-chosen by the authors for the adjusted odds ratios in the respective studies (bottom Tab. 1). Thereby, an orientating comparability of pooled adjusted *vs.* unadjusted odds ratios over all eligible studies was achieved [23]–[25].

#### Software

All calculations were performed with STATA, version 11.0, software (Stata Corp., College Station, TX, USA).

### Ethics Statement

An ethics statement was not required for this work.

## Results

### Baseline characteristics of the studies

**Figure 1:**
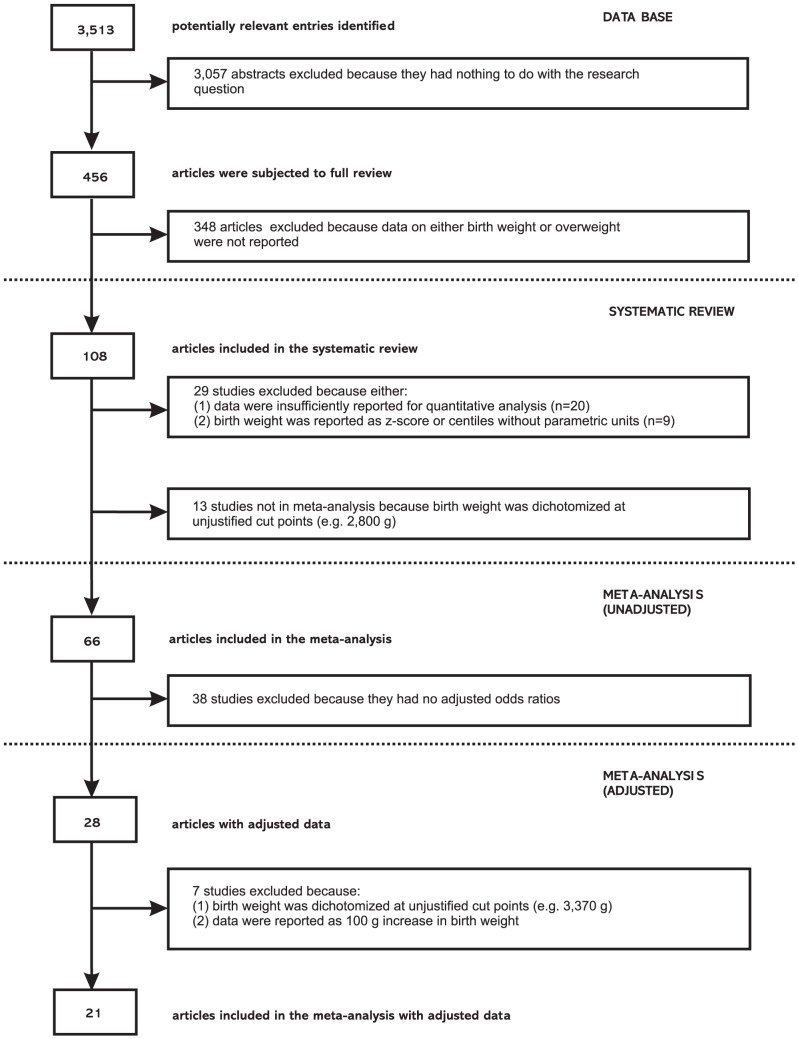
Flow diagram of selection process. Course of systematic literature review on birth weight and risk of overweight later in life, 1966–January 2011.

Course of the systematic review is illustrated in a flow diagram according to the PRISMA statement (Figure 1). From a total of 3,513 potentially relevant entries, 108 studies were identified which related birth weight to risk of later overweight [26]–[133]. They involved a total of 1,485,561 persons at six months to 79 years of age (for study characteristics see Table S1).

In the systematic review, 94 of the 108 studies (87%) reported a positive relation between birth weight and later risk of overweight. In 7 studies (6.5%), no relation between birth weight and later risk of overweight was observed, while in 6 studies (5.6%) a U-shaped relation was found. Only one study (0.9%) reported an inverse relation between birth weight and later overweight risk.

For methodological reasons, 42 studies could not be included in the meta-analysis. Reasons for exclusion were firstly, data were insufficiently reported for quantitative analysis (n = 20). Secondly, birth weight was reported as z-score or centiles without units (n = 9) and thirdly, birth weight was dichotomized at unjustified cut points (n = 13) (see Figure 1). However, these excluded studies showed no relevant differences in general characteristics, *e.g.*, distribution of geographic origin, age at follow up, assessment of overweight etc. Moreover, the observed relations between birth weight and later outcome did generally not differ from those observed in studies which could be included in the meta-analysis. Of the 42 excluded studies, 35 (83.4%) reported a positive relation between birth weight and later risk of overweight. In 3 studies (7.1%), no relation between birth weight and risk of later overweight was observed. A U-shaped relation was reported in 3 studies (7.1%) and only one study (2.4%) reported an inverse relation between birth weight and later overweight risk. These percentages were very similar to those observed in the studies which could be included into meta-analysis (see below).

For meta-analysis, 66 studies were identified to be eligible, including 58 cohort studies and eight studies with case-control-design [68]–[133], involving a total of 643,902 persons. Age of participants ranged from 1 to 75 years and the year of birth ranged from 1914–2004 (Table S1). Studies were performed in Asia, Australia, Europe, North America and South America. Study size varied ranging from 82 to 153,536 participants.

### General estimates

Of the 66 studies eligible for meta-analysis 59 studies (89.4%) reported a positive relation between birth weight and later risk of overweight. In 4 studies (6.1%), no relation between birth weight and later risk of overweight was observed, whereas in 3 studies (4.5%) a U-shaped relation was detected. None of the studies reported an inverse relation between birth weight and later overweight risk.

**Figure 2:**
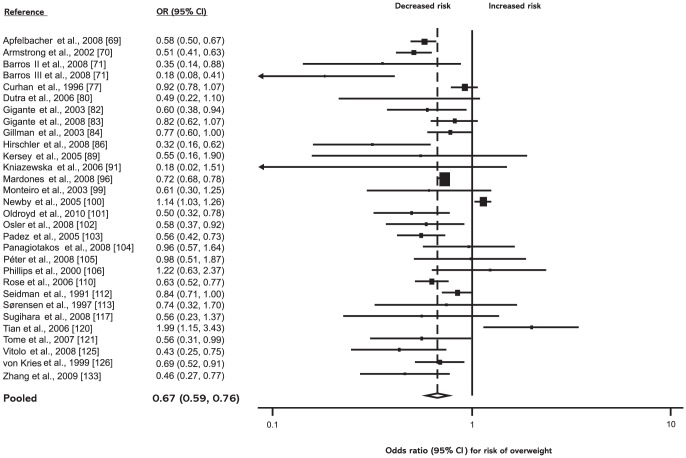
Low birth weight (<2,500 g) and subsequent risk of overweight. ORs for overweight in subjects with birth weights <2,500 g as compared with subjects with birth weights ≥2,500 g. Studies are ordered alphabetically by first author. The point estimate *(center of each black square)* and the statistical size *(proportional area of square)* are represented. Horizontal lines indicate 95% confidence intervals. The pooled odds ratio *(diamond)* was calculated by means of a random effects model. OR, odds ratio; CI, confidence interval.

*Low birth weight (<2,500 g)* was found to be associated with a decreased risk of overweight in the random-effects model (OR = 0.67; 95% CI: 0.59–0.76) as well as in the fixed-effects model (OR = 0.75; 95% CI: 0.72–0.79; see Figure 2).

**Figure 3:**
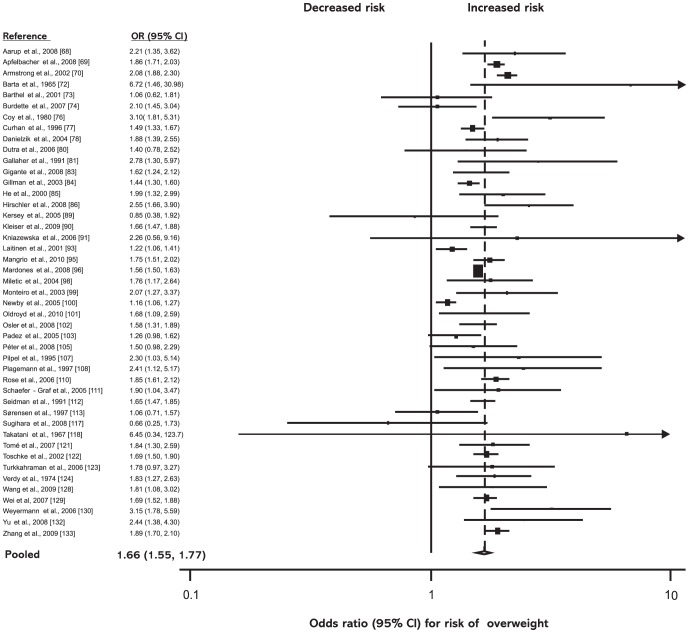
High birth weight (>4,000 g) and subsequent risk of overweight. ORs for overweight in subjects with birth weights >4,000 g as compared with subjects with birth weights ≤4,000 g. Studies are ordered alphabetically by first author. The point estimate *(center of each black square)* and the statistical size *(proportional area of square)* are represented. Horizontal lines indicate 95% confidence intervals. The pooled odds ratio *(diamond)* was calculated by means of a random effects model. OR, odds ratio; CI, confidence interval.

*High birth weight (>4,000 g)* was associated with increased risk of overweight in the random-effects model (OR = 1.66; 95% CI: 1.55–1.77) as well as in the fixed-effects model (OR = 1.61; 95% CI: 1.57–1.65; see Figure 3).

Given these results, we repeated the dichotomous comparisons, now using “normal birth weight” (2,500–4,000 g) as reference category for all studies that gave data on both ends of the birth weight spectrum. The pooled estimate for low birth weight was 0.73 (95% CI: 0.63–0.84) and those for high birth weight was 1.60 (95% CI: 1.45–1.77; all random-effects model).

*Influence analysis* showed that the pooled estimates were robust. Omission of individual studies revealed that no single study had a particular influence on the pooled estimates, detected by pooled odds ratios ranging from 0.65 (95% CI: 0.57–0.73) to 0.68 (95% CI: 0.60–0.77) for low birth weight, and 1.64 (95% CI: 1.54–1.75) to 1.68 (95% CI: 1.58–1.78) for high birth weight.

### Sensitivity/subgroup analyses

According to I^2^ [19], results were heterogeneous for both low birth weight (I^2^ = 93%) and high birth weight (I^2^ = 81%). To identify possible sources of heterogeneity and bias, we performed sensitivity/subgroup analyses (Table 1). Neither stratification by study design, nor by method of recording birth weight gave indication of recall bias. Geographic origin had no impact on the pooled estimates. Stratification by publication language also gave no indication for respective bias. Consideration of age revealed that the effect of high birth weight remained significant even in adulthood, whereas influence of low birth weight remained non-significant. Stratification by overweight classification criterion showed that studies which used non-BMI-based criteria, such as waist circumference (WC), waist-to-hip ratio (WHR) or waist-to-height ratio (WHtR), reported even stronger relations between birth weight and later overweight risk. Gender distribution had no impact on the pooled estimates. Furthermore, studies with higher lost to follow-up rates (>20%) had similar pooled estimates as those with low percentage of lost participants (≤20%). Surprisingly, a considerable number of studies did not account for gestational age, SES and/or parental body weight. However, even these potentially critical confounders had no significant impact on the overall results (Table 1).

**Table 1:** Birth weight and later risk of overweight: sensitivity and confounder analyses*.

Category	Low birth weight Odds ratio (95% CI)	High birth weight Odds ratio (95% CI)
Number of studies	n = 30	n = 45
Study design		
cohort studies	0.67 (0.59−0.76) (n = 29)	1.66 (1.56−1.78) (n = 40)
case-control studies	0.17 (0.02−1.50) (n = 1)	2.05 (1.51−2.78) (n = 5)
Geographic origin		
Europe	0.72 (0.54−0.95) (n = 11)	1.63 (1.44−1.84) (n = 20)
North America	0.76 (0.61−0.95) (n = 4)	1.64 (1.43−1.88) (n = 7)
South America	0.53 (0.42−0.68) (n = 10)	1.69 (1.48−1.93) (n = 6)
Asia	0.83 (0.48−1.42) (n = 4)	1.75 (1.62−1.89) (n = 10)
Australia	0.50 (0.32−0.77) (n = 1)	2.23 (1.22−4.06) (n = 2)
Publication language		
English	0.67 (0.59−0.77) (n = 26)	1.68 (1.57−1.80) (n = 38)
Non English	0.63 (0.43−0.94) (n = 4)	1.60 (1.30−1.97) (n = 7)
Age at follow up		
0–18 years	0.60 (0.54−0.67) (n = 23)	1.76 (1.65−1.87) (n = 37)
>18 years	0.97 (0.79−1.20) (n = 7)	1.40 (1.23−1.59) (n = 8)
Overweight classification criterion		
BMI	0.68 (0.60−0.78) (n = 25)	1.63 (1.53−1.74) (n = 36)
Non BMI	0.52 (0.24−1.11) (n = 5)	2.26 (1.85−2.75) (n = 9)
Assessment of birth weight		
registry	0.74 (0.32−1.69) (n = 1)	1.39 (0.88−2.19) (n = 2)
records/examination	0.68 (0.57−0.81) (n = 14)	1.72 (1.56−1.89) (n = 22)
interview/questionnaire	0.65 (0.53−0.80) (n = 14)	1.65 (1.48−1.83) (n = 18)
not reported	0.17 (0.02−1.50) (n = 1)	2.08 (0.69−6.19) (n = 3)
Assessment of overweight		
records/examination	0.63 (0.56−0.71) (n = 26)	1.71 (1.61−1.82) (n = 39)
interview/questionnaire	0.88 (0.70−1.11) (n = 4)	1.45 (1.24−1.71) (n = 5)
not reported	-	6.72 (1.45−31.0) (n = 1)
Gender distribution		
≤50% males	0.59 (0.41−0.85) (n = 10)	1.65 (1.43−1.91) (n = 18)
>50% males	0.70 (0.62−0.78) (n = 18)	1.71 (1.60−1.83) (n = 23)
not reported	0.66 (0.52−0.84) (n = 2)	1.68 (1.55−1.82) (n = 4)
Lost-to-follow up		
≤20%	0.57 (0.46−0.70) (n = 11)	1.72 (1.58−1.88) (n = 13)
>20%	0.74 (0.61−0.89) (n = 17)	1.65 (1.49−1.83) (n = 26)
not reported	0.53 (0.23−1.22) (n = 2)	1.80 (1.60−2.02) (n = 6)
Parental SES		
low SES>30% of population	0.71 (0.60−0.83) (n = 5)	1.64 (1.47−1.18) (n = 7)
low SES≤30% of population	0.58 (0.52−0.65) (n = 5)	1.78 (1.63−1.94) (n = 9)
not reported	0.70 (0.58−0.85) (n = 20)	1.61 (1.45−1.79) (n = 29)
Gestational age		
only term newborns	0.59 (0.41−0.84) (n = 3)	1.67 (1.44−1.94) (n = 9)
term and preterm newborns	0.65 (0.57−0.74) (n = 6)	1.70 (1.46−1.97) (n = 5)
not reported	0.68 (0.56−0.82) (n = 21)	1.66 (1.51−1.82) (n = 31)
Parental overweight		
>30% of population	0.69 (0.41−1.18) (n = 2)	1.58 (1.29−1.94) (n = 6)
≤30% of population	-	1.73 (1.55−1.93) (n = 4)
not reported	0.67 (0.58−0.76) (n = 28)	1.66 (1.54−1.79) (n = 35)
***Confounder-adjusted analyses***		
*Studies with adjustments*		
*unadjusted estimates*	*0.50 (0.32−0.78) (n = 1)*	*1.87 (1.56−2.25) (n = 16)*
*adjusted estimates*	*0.51 (0.33−0.80) (n = 1)*	*1.93 (1.56−2.38) (n = 16)*
*Studies without adjustments*	*0.68 (0.60−0.77) (n = 29)*	*1.68 (1.54−1.84) (n = 29)*

Abbreviation: BMI, body mass index; CI, confidence interval

&ast; random-effects model

### Impact of publication bias

Neither for the relation between low birth weight and risk of overweight nor for that between high birth weight and later overweight risk evidence for publication bias was found, as indicated by visual inspection of funnel plots, proven by nonsignificant Begg's tests (low birth weight: *p* = 0.80; high birth weight: *p* = 0.45) and Egger's tests (low birth weight: *p* = 0.07; high birth weight: *p* = 0.23).

### Shape of the continuous association between birth weight and risk of overweight

**Figure 4:**
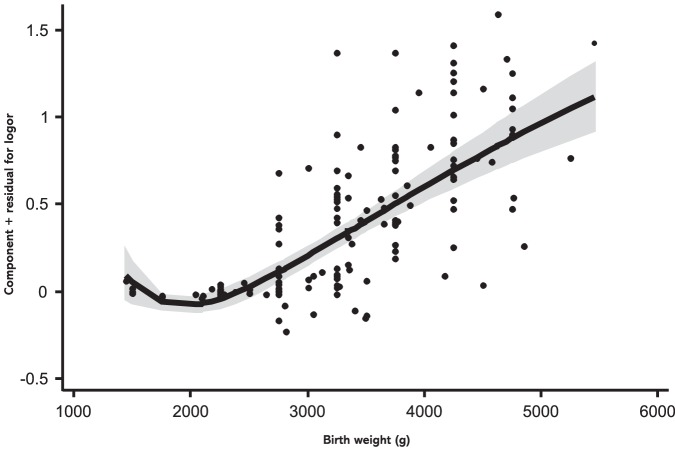
Relationship between birth weight and risk of overweight. Continuous relation between birth weight and later risk of overweight, calculated by fractional polynomial regression. Studies are represented by black dots. Grey shading indicates the 95% confidence interval around the fitted line. The model was estimated from a robust regression model based on second-order fractional polynomial (−1, −0.5) functions weighted by/variance.

In a first step, we fitted a linear meta-regression model to the data, which revealed a significant positive linear relation between birth weight and subsequent overweight risk (b = 0.34×10^−3^ (0.28−0.40×10−3); *p*<0.001). To further explore the shape of the association, fractional polynomial regression was used. Figure 4 shows the shape of the curve for the continuous relation between birth weight and later risk of overweight, estimated by a second-order fractional polynomial regression model (b_1_ = 14.13 (9.27–19.00); b_2_ = −20.34 (−26.58−(−14.09)); p_1_ = −1; p_2_ = −0.5; *p*<0.001; inverse variance weighted). Over nearly the entire birth weight spectrum, birth weight was found to be linearly positively related to overweight risk. Risk does not further decrease below a birth weight of 1,500 g. Comparison of model fitness parameters showed that a linear regression model performed as good as the fractional polynomial model (*p* = 0.17 for comparison of deviance between both models).

### Impact of confounders on strength of the association

Whereas only one study gave an adjusted estimate for low birth weight, in 16 studies confounder-adjusted estimates for risk of overweight after high birth weight were reported. Number and types of adjustments varied across the studies (Table S2). The pooled confounder-adjusted estimate for overweight after high birth weight was nearly the same as the pooled estimate of the unadjusted data from the same studies using the same reference category (Table 1). Fully adjusted estimates revealed a nearly doubled long-term overweight risk in individuals with high birth weight compared to normal birth weight subjects (OR = 1.96; 95% CI: 1.43–2.67). Weighted fractional polynomial regression of the confounder-adjusted data revealed very similar results as in the case of unadjusted data (b_1_ = 16.59 (9.55–23.64); b_2_ = −24.11 (−33.06 (−15.16)); p_1_ = −1; p_2_ = −0.5; *p*<0.001; inverse variance weighted).

## Discussion

Over several years, increasing attention is given on early developmental origins of long-term diabetic, adipogenic, and cardiovascular disorders in terms of the metabolic syndrome [10], [11]. However, the direction and strength of relation between birth weight and long-term overweight risk has not been tested globally so far in a comprehensive manner. Therefore, we aimed to characterize this overall critical aspect of the ‘fetal origins’ approach by a respective meta-analysis, quantitatively as well as qualitatively substantially extending and proving previous preliminary reviews, statements, proposals etc. by our own group and others [15]–[17].

The data set and meta-analysis provided here is by far the largest one analyzed to date on this topic and the first to address the whole lifespan, provide confounder-adjusted estimates and incorporate global data *i.e*., including western, westernized as well as developing countries in Northern- and South America, Europe, Asia and Australia. Our analysis shows with high consistency that an increased birth weight (>4,000 g) may lead to a doubling of the long-term overweight risk, irrespective of geographic/ethnic origin, sex, socio-economic status, parental weight status etc.

In the context of the ‘small baby syndrome’ and the respective ‘fetal origins’ hypotheses [10]–[12], it has been claimed that low birth weight is a risk factor for cardiovascular diseases and type 2 diabetes, as tested by systematic reviews and meta-analyses with mixed results [134]–[138], as well as for the development of overweight/obesity, *i.e.*, one of the most critical cardiometabolic risk determinants [5]–[7]. Interestingly, results of our meta-analysis do not support this claim. In contrast, across the birth weight spectrum a linear positive relation exists with later overweight risk. Only a small fraction of studies, analyzing probands with very low birth weight (VLBW; <1,500 g; n = 3 studies), found no further decrease of overweight risk at this very ‘left-handed’ side of the birth weight spectrum.

General concerns on our data and their interpretation might arise regarding the suitability of investigated parameters and/or the consideration of bias and/or confounding variables. For instance, it has increasingly been proposed that the effect of low birth weight on later health risks might rather result from increased (‘rapid’) neonatal weight gain/catch up growth [139]. This was regarded, unfortunately, in only five reports (4.6%; Table S3) [79], [109], [114], [115], [116], three of which [79], [115], [116] reported respectively adjusted data analyses. An ‘obesogenic’ effect of low birth weight was not observed in these unadjusted or adjusted studies. By contrast, all found an effect of high birth weight on later overweight risk, independently of early weight gain. Furthermore, although BMI has been established as routine parameter to identify overweight, it does not necessarily describe the cardiometabolically critical fat content and distribution [140]. Alternative measures, particularly reflecting abdominal obesity (waist circumference, waist-to-hip ratio, waist-to-height ratio), have been shown to be more accurate risk predictors [140]. Respective subgroup analyses revealed, however, that the relation between birth weight and later overweight risk was even strengthened when non-BMI related measures were applied. Also subgroup analyses considering gestational age, age at follow-up, geographic origin etc. showed no significant influence on the overall outcome. Moreover, pooled adjusted estimates were calculated considering the influence of confounders on the strength of the relation under investigation. However, they had no significant impact on the final outcome. Even parental weight status had only a marginal influence on the relation between birth weight and later overweight risk. This result appears to be of particular interest, since parental weight/overweight might represent contribution of genetic factors to the investigated relationship. Interestingly, it has been shown that none of the genetic obesity risk factors identified to date has a significant influence on birth weight [141], making respective confounding rather unlikely, as supported here.

Recently, Yu et al. [17] published a systematic review on the association between birth weight and downstream obesity, including 129,260 subjects from 33 studies, mainly originating from Asia (58%). Their meta-analysis involved 20 studies with a total of 42,863 subjects, most from China (15 studies; 88% of subjects). We analyzed a similar number of studies from Asia (n = 12) with a higher number of subjects from Asia (n = 140,734) as well as China (n = 103,411) in our meta-analysis. Interestingly, focussing on Asian/Chinese subjects Yu et al. [17] came to similar results as presented here: low birth weight was accompanied by decreased risk of obesity later on (OR = 0.61; 95% CI: 0.46–0.80), while high birth weight was associated with increased obesity risk (OR = 2.07; 95% CI: 1.91–2.24). Their analysis focused solely on obesity, with the meta-analysis only on children and adolescents including mainly Asian/Chinese subjects, studies and databases. A number of variables (*e.g.*, geographic origin/ethnicity, gestational age, age at follow-up, parental weight etc.) were not considered and confounder adjusted estimates were not provided. Nevertheless, the observed trend in the relation under investigation was similar to those ascertained and described in our study. This appears to underline the global impact and reproducibility of the observed relationship and indicates that it is even relevant for Asian populations, characterized by a high frequency of relatively ‘low birth weight’ subjects but, simultaneously, continuous increase of ‘diabesity’ prevalence [142], [143].

Therefore, it must be noted that the provided data analyses should not be interpreted in terms of a ‘beneficial’ effect of a reduced birth weight. To the contrary, epidemiological, clinical as well as experimental observations have convincingly demonstrated long-term deleterious consequences in association with a decreased birth weight, especially concerning metabolic-syndrome-like disorders and diseases [10], [11]. Therefore, future studies should consider more precise measures of body composition, fat content and, especially, accompanying metabolic and hormonal alterations both at birth and later life to better understand pathophysiological links between altered prenatal nutritional and growth conditions and later overweight and ‘diabesity’ risk. Future studies should generally consider important variables in the relationship between fetal growth and later outcome, especially the potential impact of gestational age, maternal diseases during pregnancy, as well as neonatal nutrition, growth pattern and fat deposition. Finally, a slight trend has been observed here towards successively increasing risk of overweight in low birth weight subjects with increasing age, *i.e.*, in adulthood (Table 1). Therefore, a ‘U-shaped’ curve, as it has been described regarding type 2 diabetes and hypertension [136], [134], with increasing age up to the elderly of the relation between birth weight and later risk of overweight, cannot be excluded from our data. Occasionally, pathophysiological causes and mechanisms of a latency of overweight manifestation in formerly low birth weight subjects remain to be evaluated.

Birth weight is essentially determined by the *in-utero* developmental conditions (13, 14), especially the materno-fetal food supply [144]–[146]. Accordingly, it appears important to notice that in parallel with the global ‘diabesity’ epidemics [2], [3]; the number of overweight and/or diabetic women at reproductive age has increased dramatically [147], [148]. Overweight and/or diabetes during pregnancy, however, lead to fetal overnutrition, often followed by increased birth weight, fatness and macrosomia at birth [144]–[146], [149], [150]. All of this has been shown to be preventable by adequate nutritional and metabolic management during pregnancy [151], [152]. With respect to our data of increased overweight risk in formerly macrosomic newborns, respective preventive measures may therefore not only improve the peripartal and perinatal outcome [151], [152], but even the long-term overweight risk and resulting disease dispositions. This prediction is in line with a number of epidemiological, clinical and experimental data which have shown an increased risk of overweight, obesity and diabetes in offspring of diabetic and/or obese pregnant women, even independent of or in addition to genetic dispositions [153]–[157]. Especially, fetal hyperglycemia/overnutrition leads to fetal B cell hyperplasia and hyperinsulinism, which have been shown to be preserved for the long term and subsequently may predispose to insulin resistance and a permanent obesity disposition [144]–[146], [158]–[161]. In general, mechanistic approaches speak in favour of epigenomic and/or microstructural long-term malprogramming of body weight regulatory systems by fetal overfeeding and accompanying hormonal disturbances during critical periods of fetal development, predisposing to increased overweight risk for the long-term [146], [154], [157]–[162].

Accordingly, prenatal life appears to be a ‘critical period’ [163], [164] of determination and, consequently, potential genuine prevention of long-term overweight predisposition and its critical co-morbidity [165]. Interestingly, high birth weight has also been described to be a risk factor for, *e.g.*, type 2 diabetes, hypertension, childhood primary brain tumors and breast cancer [136], [134], [166], [167], all shown to be critically linked to overweight throughout life [2]–[4], [168].

Taken together, increased birth weight is reproducibly and independently linked to increased overweight risk later on, suggesting prenatal overfeeding as important risk factor which ‘programms’ a long-term obesity predisposition. In conclusion, avoiding *in-utero* overnutrition, especially by avoiding and/or adequately managing maternal overweight, overnutrition, increased weight gain and/or diabetes during pregnancy, appears to be a promising strategy to lower overweight risk for the long term, globally.

## Supporting Information

Checklist S1 PRISMA checklist.
(DOC)

Protocol S1 Study protocol for systematic review and meta-analysis to determine the relation between birth weight and long-term overweight risk.
(DOC)

Table S1 Characteristics of 108 studies included in the systematic review of birth weight and subsequent risk of overweight, 1966–January 2011.
(DOC)

Table S2 Studies that adjusted for confounders in the meta-analysis on birth weight and subsequent risk of overweight, 1966-January 2011.
(DOC)

Table S3 Studies that reported data on neonatal weight gain or infant growth in the meta-analysis on birth weight and subsequent risk of overweight, 1966-January 2011.
(DOC)
