# Supplementary material for: Birth Weight and Long-Term Overweight Risk: Systematic Review and a Meta-Analysis Including 643,902 Persons from 66 Studies and 26 Countries Globally
Source: PLoS One. 2012 Oct 17;7(10):e47776. doi: 10.1371/journal.pone.0047776 (PMC3474767; doi:10.1371/journal.pone.0047776)
Supplement: Protocol S1 — Study protocol for systematic review and meta-analysis to determine the relation between birth weight and long-term overweight risk. (DOC) [file pone.0047776.s002.doc]

**Protocol S1** Study protocol for systematic review and meta-analysis to determine the relation between birth weight and long-term overweight risk.

Schellong et al. **“Birth weight and long-term overweight risk: systematic review and a meta-analysis including 643,902 persons from 66 studies and 26 countries globally”**

**Objective**

To examine the direction and strength of relation between birth weight and long-term risk of overweight.

**Inclusion criteria**

*Study type*

- All study types will be eligible to enter meta-analysis.
- We will include all studies which were published as original report and present information on the relation between birth weight and long-term overweight risk.

*Participants*

- Subjects of all age groups will be included.
- If data for overweight at different age groups were reported we will extract data at the highest age group.
- If possible, we will perform sub-group analyses (e.g. different age groups, geography, etc.).
- Even studies which reported relation between birth weight and long-term overweight risk as side effect will be included.

*Definition of exposition*

- All studies which reported information on birth weight in at least to strata of birth weight will be included as well as studies which reported odds ratios (OR) and 95% confidence intervals (95% CI) (or data with which to calculate them) for risk of overweight per unit of birth weight.
- Within individual birth weight strata we accept variations up to 100g of birth weight (e.g. in the category <2,500g vs. >2,500g we will tolerate <2,401g vs. >2,599g as well).

*Outcome variable*

- All studies which reported the proportion of overweight subjects in at least one age at follow up will be included.
- We will not restrict to a particular definition of overweight as studies may have been published before currently accepted definitions were introduced*.*
- If studies report data for overweight as well as obesity, we will use the data with the lowest definition.

Outcome measures

- The OR will either be extracted from the published article or calculated by the reviewers.
- If the OR is not directly reported or cannot be readily extracted from the published data, the reviewers will contact the corresponding authors for additional information (e.g., data provided in 2x2 contingency tables).

*Publication type*

- Full published papers will be eligible (no language restrictions).

**Search Methods**

We will search the following electronic databases:

- MEDLINE
- EMBASE

There will be no restriction on language or year of publication. In these databases, we will search according to the thesaurus of the NCBI MESH browser the following terms and combinations of keywords in full text:

The following keywords will be employed:

1. “birth weight”
2. “overweight”
3. “obes*” (truncated)
4. “adipos*” (truncated)
5. “1” and (“2” OR “3” OR “4”)

Additionally, bibliographies of identified publications and published reviews will be hand searched for potentially relevant articles. Authors will be contacted if data, methods and/or parameter definitions provided from the respective studies are unclear.

**Reviews**

All references cited in the identified reviews will be manually searched for potentially relevant studies.

**Data collection**

Three reviewers (KS, SS, TH) will independently scrutinize the list of titles, and if available the abstracts, to determine potential usefulness of the article. Final selection will be based on the full text of potentially relevant articles by the three reviewers independently. In cases of disagreement, a fourth reviewer (AP) will examine such articles. Results will be discussed until reaching consensus among all four reviewers.

The following study characteristics will be extracted: publication year, country, study design, year of birth, age at outcome, lost to follow-up, cohort size, assessment of birth weight, assessment of overweight, overweight criterion, sex, main result.

From all eligible studies, relevant data will be abstracted in duplicate, using a standardized data extraction sheet. An independent reviewer will confirm all data entries and will check at least twice for completeness and accuracy.

**Meta-analysis**

*Dichotomous comparisons*

- Data on numbers of subjects with and without overweight above or below the cutoff value and corresponding crude odds ratios and 95% confidence intervals will be calculated.
- Fixed-effects as well as random-effects models to estimate the pooled odds ratios for risk of overweight above *vs.* below the respective cutoff value will be constructed across all studies.

*Strategies for evaluation*

1. Birth weight >4,000g vs. <4,000g*
2. Birth weight <2,500g vs. > 2,500g
3. Birth weight >4,000g vs. (<4,000g but >2,500g)
4. Birth weight <2,500g vs. (>2,500g but <4,000g)

*Some authors define macrosomia at >4,500g. These studies will be accepted as well.

*Assessment of heterogeneity*

- Impact of heterogeneity will be assessed by calculating the I^2^ according to Higgins et al (Higgins JP et al. 2003).

*Influence analysis*

- Robustness of the pooled estimates will be checked by influence analyses. Each of the studies will be individually omitted from the data set, followed in each case by recalculation of the pooled estimate of the remaining studies.

*Meta-regression*

- To explore the shape of the continuous relation between birth weight and later overweight risk, meta-regression technique will be applied (Berlin JA et al. 1993).

*Subgroup/Sensitivity analyses*

- To identify potential sources of heterogeneity and sources of bias, studies will be stratified by study design and assessment of birth weight data to assess potential recall bias.
- Studies will be stratified by publication language.
- To examine participation/selection bias, we will stratify by extent of lost-to-follow-up (<20% vs. >20%).
- Further stratifications will be made by geographic origin, age at follow up, overweight classification criterion, assessment of overweight, gender distribution (<50% males vs. >50% males), parental SES (> 30% vs. <30%), gestational age (only term newborns vs. term and preterm newborns) and parental overweight (BMI > 25 kg/m²).

**Evaluation of bias and confounding**

*Language bias*

- Pooled estimates of all English-language studies will be compared with that of all non-English language studies.

*Publication bias*

- Publication bias will be assessed by inspection of the funnel plot and formal testing for funnel plot asymmetry, using Begg’s test and Egger’s test (Sterne JA et al. 2001).

*Analysis of confounder-adjusted data*

- To perform meta-analysis of confounder-adjusted data, we will consider all studies which report adjusted odds ratios for risk of overweight for the birth weight categories <2,500 g and/or >4,000 g.

**Discussion and Evaluating**

- The results will be critically and integratively discussed.

**References**

Higgins JP, Thompson SG, Deeks JJ, Altman DG (2003) Measuring inconsistency in meta- analysis. BMJ 327: 557-560.

Berlin JA, Longnecker MP, Greenland S (1993) Meta-analysis of epidemiologic dose- response data. Epidemiology 4: 218-228.

Sterne JA, Egger M, Smith GD (2001) Systematic review in health care: investigating and dealing with publication and other biases in meta-analysis. BMJ 323: 101-105.
