# Supplementary material for: Birth Weight and Long-Term Overweight Risk: Systematic Review and a Meta-Analysis Including 643,902 Persons from 66 Studies and 26 Countries Globally
Source: PLoS One. 2012 Oct 17;7(10):e47776. doi: 10.1371/journal.pone.0047776 (PMC3474767; doi:10.1371/journal.pone.0047776)
Supplement: Table S1 — Characteristics of 108 studies included in the systematic review of birth weight and subsequent risk of overweight, 1966–January 2011. (DOC) [file pone.0047776.s003.doc]

**Table S1 Characteristics of 108 studies included in the systematic review of birth weight and subsequent risk of overweight, 1966 – January 2011**

| **Reference** | | **Country** | **Design** | **Year of birth** | **Age at** | **Lost to** | **Cohort size** |
| --- | --- | --- | --- | --- | --- | --- | --- |
|  | |  |  |  | **Outcome** | **follow - up** | **(final)** |
| Aarup et al,  2008 [68] | | Denmark | CO | 1999 | 3 years | 1.45% | 813 |
| Apfelbacher et al, 2008 [69] | | Germany | CO | 1984 - 1995 | 5 - 7 years | 21% | 34,809 |
| Araújo et al,  2008 [26] | | Brazil | CO | 1995 - 1996 | 11 years | 13% | 5,160 |
| Armstrong et al,  2002 [70] | | United Kingdom | CO | 1995 - 1996 | 39 - 42 months | 39% | 32,200 |
| Barros (I) et al,  2008 [71] | | Brazil | CO | 1982 | 12 month | 21% | 1,441 |
| Barros (II) et al,  2008 [71] | | Brazil | CO | 1993 | 12 month | 7% | 1,361 |
| Barros (III) et al,  2008 [71] | | Brazil | CO | 2004 | 12 month | 6% | 3,906 |
| Barta et al,  1965 [72] | | Hungary | CC | 1947 - 1957 | 8 years | 85% | 598 |
| Barthel et al,  2001 [73] | | France | CO | not reported | 10 years | not reported | 3,621 |
| Binkin et al,  1988 [27] | | USA | CO | 1975 - 1984 | 36 - 41 months | not reported | 437,431 |
| Boney et al,  2005 [28] | | USA | CC | not reported | 11 years | 91% | 109 |
| Bouhours - Nouet et al,  2008 [29] | | France | CO | 1986 - 1998 | 6 - 15 years | 69% | 117 |
| Burdette et al,  2007 [74] | | USA | CO | 1998 - 2000 | 3 years | 13% | 2,133 |
| Celi et al,  2003 [30] | | Italy | CO | not reported | 3 - 17.5 years | 73% | 12,143 |
| Charney et al,  1976 [31] | | USA | CO | 1945 - 1955 | 20 - 30 years | 15% | 344 |
| Chen et al,  2006 [75] | | USA | CO | 1959 - 1965 | 8 years | 41% | 34,866 |
| Coy et al,  1973 [76] | | Australia | CO | 1967 - 1968 | 1 year | 8% | 1,022 |
| Curhan et al,  1996 [77] | | USA | CO | 1928 (median) | 40 - 75 years | 56% | 22,846 |
| da Costa Ribeiro  et al, 2003 [32] | | Brazil | CC | not reported | 7 - 10 years | 82% | 446 |
| Danielzik et al,  2004 [78] | | Germany | CO | not reported | 5 - 7 years | 47% | 2,631 |
| de Lourdes Drachler et al,  2003 [33] | | Brazil | CO | not reported | 12 - 59 months | 26% | 2,569 |
| de Moraes et al,  2006 [34] | | Mexico | CO | not reported | 9 - 9.7 years | 5% | 662 |
| Dennison et al,  2006 [79] | | USA | CO | not reported | 4 years | 78% | 616 |
| Dieu et al,  2007 [35] | | Vietnam | CO | 2000 | 4 - 6 years | not reported | 670 |
| Dubois et al,  2006 [36] | | Canada | CO | 1998 | 44 - 56 months | 31% | 1,450 |
| Dutra et al,  2006 [80] | | Brazil | CO | not reported | 10 - 19 years | 2% | 810 |
| Eriksson et al,  2001 [37] | | Finland | CO | 1924 - 1933 | 7 years | 48% | 3,659 |
| Frye et al,  2003 [38] | | Germany | CO | 1992 - 1999 | 5 - 14 years | 18% | 6,263 |
| Fuiano et al,  2008 [39] | | Italy | CO | 1997 - 2002 | 3 - 6 years | not reported | 632 |
| Gallaher et al,  1991 [81] | | USA | CO | 1983 or later | 1 - 5 years | 39% | 261 |
| Gigante et al,  2003 [82] | | Brazil | CO | 1993 | 1 - 4 years | 13% | 1,273 |
| Gigante et al,  2008 [83] | | Brazil | CO | 1982 | 22 - 23 | 29% | 4,197 |
| Gillman et al,  2003 [84] | | USA | CO | not reported | 9 - 14 years | 44% | 14,881 |
| Guimarães et al,  2006 [40] | | Portugal | CC | not reported | 6 - 11 years | 68% | 430 |
| Hack et al,  2003 [41] | | USA | CO | 1977 - 1979 | 20 years | 53% | 403 |
| Hawkins et al,  2008 [42] | UK | | CO | 2000 - 2002 | 9.2 month,  3 years | 28%  20% | 13,043 |
| He et al,  2000 [85] | China | | CC | 1986 - 1996 | 3 - 6.9 years | 31% | 919 |
| Hirschler et al,  2008 [86] | Argentina | | CO | 1993 - 2002 | 5 - 13 years | 18% | 1,027 |
| Hui et al,  2003 [87] | China | | CC | not reported | 6 - 7 years | 12% | 341 |
| Hui et al,  2008 [43] | China | | CO | 1997 | 7 years | 22.5% | 6,075 |
| Kang et al,  2006 [88] | Korea | | CO | not reported | not reported | 10% | 3,627 |
| Kersey et al,  2005 [89] | USA | | CO | not reported | 2 - 5 years | 28% | 364 |
| Kleiser et al,  2009 [90] | Germany | | CO | 1986 - 2003 | 3 - 17 years | 27.4% | 12,793 |
| Kniażewska et al,  2006 [91] | Poland | | CC | not reported | 6 - 17 years | not reported | 82 |

| Koupil et al,  2008 [44] | Sweden | CO | 1973 - 1985 | 18.2 years | 19% | 6,535 |
| --- | --- | --- | --- | --- | --- | --- |
| Kromeyer - Hausschild et al,  1999 [92] | Germany | CO | not reported | 7 - 14 years | 81% | 1,031 |
| Laitinen et al,  2001 [93] | Finland | CO | 1965 - 1967 | 31 years | 49% | 6,280 |
| Leong et al,  2003 [45] | USA | CO | not reported | 50 - 79 years | 0.1% | 1,850 |
| Li et al,  2007 [46] | USA | CO | 1984; 1986;  1988; 1990 | ≤12 years | 85% | 1,739 |
| Locard et al,  1992 [47] | France | CC | not reported | 5 years | 18% | 1,031 |
| Lundgren et al,  2003 [48] | Sweden | CO | 1973 - 1983 | 16 - 26 years | 1% | 43,872 |
| Maddah et al,  2008 [49] | Iran | CO | 1988 - 1992 | 14 - 17 years | 10% | 2,090 |
| Maffeis et al,  1994 [94] | Italy | CO | not reported | 4 - 12 years | 11% | 1,363 |
| Mangrio et al,  2010 [95] | Sweden | CO | 1999 - 2004 | 4 years | 1.37% | 8,885 |
| Mardones et al,  2008 [96] | Chile | CO | 1997 - 1999 | 6 - 8 years | 8% | 153,536 |
| Mazur et al (1),  2003 [50] | Poland | CO | not reported | urban: 6.6 - 15.1 years  rural: 6.8 - 14.5 years | 0% | 2,066 |
| Mazur et al (2),  2003 [51] | Poland | CO | not reported | urban: 6.9 - 14.9 years  rural: 6.9 - 14.7 years | 0.5% | 2,181 |
| Mazur et al,  2008 [52] | Poland | CO | not reported | girls: 10.4 years  boys: 10.5 years | 18% | 4,248 |
| Meas et al,  2008 [53] | France | CO | 1971 - 1985 | 22 and 30 years | 24% | 851 |
| Mikulandra et al,  2000 [97] | Croatia | CC | not reported | 27 - 31 years | not reported | 1,987 |
| Miletić et al,  2004 [98] | Croatia | CO | not reported | 27.9 years  (mean) | not reported | 735 |
| Mogan et al,  1986 [54] | Canada | CO | not reported | 6 months | 21% | 62 |
| Monteiro et al,  2003 [99] | Brazil | CO | 1982 - 1984 | 14 - 16 years | 82% | 1,073 |
| Newby et al,  2005 [100] | Sweden | CO | 1914 - 1948 | 60 years | 80% | 18,109 |
| O’Callaghan et al,  1997 [55] | Australia | CO | 1981 - 1984 | 5 years | 45% | 4,061 |
| Ochoa et al,  2007 [56] | Spain | CC | not reported | 6 - 18 years | not reported | 370 |
| Oldroyd et al,  2010 [101] | Australia | CO | 1999 and 2000 | 4 - 5 years | 48% | 4,369 |
| Olson et al,  2008 [57] | USA | CO | not reported | 3 years | 66% | 208 |
| Osler et al,  2008 [102] | Denmark | CO | 1953 | 19 years | 21% | 9,143 |
| Padez et al,  2005 [103] | Portugal | CO | not reported | 7 - 9.5 years | 36% | 4,415 |
| Panagiotakos et al,  2008 [104] | Greece | CO | 1993 - 1995 | 10 - 12 years | 43% | 400 |
| Péter et al,  2008 [105] | Hungary | CO | 1986 - 1999 | 7 - 19 years | 0% | 1,334 |
| Phillips et al,  2000 [106] | United Kingdom | CO | 1920 - 1930 | 59 - 73 years | 22% | 1,750 |
| Pilpel et al,  1995 [107] | Israel | CO | not reported | 17 - 18 years | not reported | not reported |
| Plagemann et al,  1997 [108] | Germany | CO | 1980 - 1990 | 1 - 9 years | 44% | 178 |
| Reilly et al,  2005 [109] | United Kingdom | CO | 1991 - 1992 | 7 years | 44% | 7,758 |
| Rose et al,  2006 [110] | USA | CO | not reported | 6.2 years | not reported | 16,889 |
| Rugholm et al,  2005 [58] | Denmark | CO | 1936 - 1983 | 13 years | not reported | 252,961 |
| Savva et al,  2005 [59] | Cyprus | CO | not reported | 2 - 6.9 years | 29% | 1,412 |
| Schaefer-Graf et al,  2005 [111] | Germany | CO | 1995 - 2000 | 5.4 years | 58% | 324 |
| Seidman et al,  1991 [112] | Israel | CO | 1964 - 1971 | 17 years | 1% | 33,413 |
| Seidman et al,  1998 [60] | Israel | CO | 1974 - 1976 | 17 years | 6% | 10,804 |
| Serra - Majem et al,  2006 [61] | Spain | CO | not reported | 2 - 13 years | not reported | 1,375 |
| Shehadeh et al,  2008 [62] | Israel | CC | not reported | 12 years | not reported | 302 |
| Sørensen et al,  1997 [113] | Denmark | CO | after 1973 | 20 years | 11% | 4,300 |
| Stettler (1) et al,  2002 [114] | Seychelles | CO | not reported | 4.5 - 17.4 years | 17% | 5,514 |
| Stettler (2) et al,  2002 [115] | USA | CO | 1959 - 1965 | 7 years | 31% | 19,397 |
| Stettler et al,  2003 [116] | USA | CO | 1962 - 1966 | 20 years | 33% | 300 |
| Strufaldi et al,  2008 [63] | Brazil | CO | 1996 - 2000 | 6 - 10 years | 23% | 739 |

| Sugihara et al,  2008 [117] | Japan | CO | 1986 - 1994 | 9 - 17 years | 25% | 195 |
| --- | --- | --- | --- | --- | --- | --- |
| Sugimori et al,  1997 [64] | Japan | CC | 1989 | 3 years | 89% | 351 |
| Takahashi et al,  1999 [65] | Japan | CC | 1989 | 3 years | 87% | 1,281 |
| Taketani et al,  1967 [118] | Japan | CC | 1962 - 1963 | 3 years | 95% | 88 |
| Tene et al,  2003 [119] | Mexico | CC | not reported | not reported | 15% | 322 |
| Terry et al,  2007 [66] | USA | CO | 1959 - 1965 | 40 years | 68% | 261 |
| Tian et al,  2006 [120] | China | CO | not reported | 18 - 74 years | 56% | 973 |
| Tomé et al,  2007 [121] | Brazil | CO | 1978 - 1979 | 8 - 10 years | 59% | 2,796 |
| Toschke et al,  2002 [122] | Czech Republic | CO | not reported | 6 - 14 years | 2% | 33,768 |
| Turkkahraman et al,  2006 [123] | Turkey | CO | not reported | 6 - 17 years | 23% | 1,891 |
| Verdy et al,  1974 [124] | Canada | CO | not reported | not reported | not reported | 734 |
| Vitolo et al,  2008 [125] | Brazil | CO | 1997 | 1 month - 5 years | 6% | 3,714 |
| Vohr et al,  1980 [67] | USA | CO | not reported | 7 years | 37% | 33 |
| von Kries et al,  1999 [126] | Germany | CO | not reported | 5 - 6 years | 93% | 9,206 |
| von Kries et al,  2002 [127] | Germany | CO | not reported | 5 - 6.99 years | 12% | 6,483 |
| Wang et al,  2009 [128] | China | CC | 1993 - 1995 | 10 - 14 years | 4.5% | 2,870 |
| Wei et al,  2007 [129] | China | CO | not reported | 6 - 18 years | 57% | 81,538 |
| Weyermann et al,  2006 [130] | Germany | CO | 2000 - 2001 | 2 years | 46% | 855 |
| Ylihärsilä et al,  2007 [131] | Finland | CO | 1934 - 1944 | 61.5 years | 77% | 2,000 |
| Yu et al,  2008 [132] | China | CO | 1998 - 2001 | 1 - 3 years | 54% | 918 |
| Zhang et al,  2009 [133] | China | CO | not reported | 3 - 6 years | 4% | 15,852 |

**Table S1 [continued]**

| **Reference** | **Assessment of**  **birth weight** | **Assessment of**  **overweight** | **Obesity Criterium** | **Sex** | **Main result** | **Reasons for exclusion from meta-analysis** | **Remarks** |
| --- | --- | --- | --- | --- | --- | --- | --- |
| Aarup et al,  2008 [68] | questionnaire | questionnaire | BMI | 49% males | positive * |  |  |
| Apfelbacher et al,  2008 [69] | questionnaire | examination | BMI | 51% males | positive * |  |  |
| Araújo et al,  2008 [26] | examination | examination | BMI | not reported | positive * | birth weight dichotomized  at unjustified cut points |  |
| Armstrong et al,  2002 [70] | records | records | BMI | 50% males | linear positive | birth weight dichotomized  at unjustified cut points |  |
| Barros (I) et al,  2008 [71] | examination | examination | z - score of  weight for length >2  standard deviations | 49% males | linear positive |  |  |
| Barros (II) et al,  2008 [71] | examination | examination | z - score of  weight for length >2  standard deviations | 49% males | linear positive |  | 1993: cohort identical to Araújo et al, 2008 |
| Barros (III) et al,  2008 [71] | examination | examination | z - score of  weight for length >2  standard deviations | 52% males | linear positive |  |  |
| Barta et al,  1965 [72] | not reported | not reported | not reported | 49% males | positive * |  |  |
| Barthel et al,  2001 [73] | records | examination | BMI | 50% males | positive * |  |  |
| Binkin et al,  1988 [27] | records | records | weight - for - height | not reported | linear positive | insufficient data for  quantitative analysis |  |
| Boney et al,  2005 [28] | records | examination | BMI | 48% males | positive * | birth weight was reported  as centile without units |  |
| Bouhours-Nouet  et al, 2008 [29] | records | examination | BMI - SDS | 41% males | positive | insufficient data for  quantitative analysis (birth weight as centiles) |  |
| Burdette et al,  2007 [74] | not reported | examination | BMI | 52% males | positive * |  |  |
| Celi et al,  2003 [30] | questionnaire | examination | BMI | 49% males | positive * | birth weight dichotomized  at unjustified cut points |  |
| Charney et al,  1976 [31] | records | questionnaire | weight - for - height | not reported | positive * | birth weight was reported  as centile without units |  |
| Chen et al,  2006 [75] | not reported | examination | BMI | 50% males | linear positive |  |  |
| Coy et al,  1973 [76] | records | records | weight percentiles | 50% males | linear positive |  |  |
| Curhan et al,  1996 [77] | questionnaire | questionnaire | BMI | 100% males | linear positive |  |  |
| da Costa Ribeiro  et al, 2003 [32] | not reported | examination | weight - for - height | 48% males | positive * | birth weight dichotomized  at unjustified cut points |  |

| Danielzik et al,  2004 [78] | records | examination | BMI | 49% males | linear positive |  |  |
| --- | --- | --- | --- | --- | --- | --- | --- |
| de Lourdes Drachler et al, 2003 [33] | records | examination | weight - for - height | not reported | linear positive | birth weight was reported  as centile without units |  |
| de Moraes et al,  2006 [34] | records | examination | BMI | 50% males | linear positive | insufficient data for  quantitative analysis |  |
| Dennison et al,  2006 [79] | records | examination | BMI | 51% males | linear positive |  |  |
| Dieu et al,  2007 [35] | questionnaire | examination | BMI | 50% males | linear positive | insufficient data for  quantitative analysis |  |
| Dubois et al,  2006 [36] | questionnaire | examination | BMI | 51% males | U - shaped | insufficient data for  quantitative analysis |  |
| Dutra et al,  2006 [80] | interview | examination | BMI | 50% males | linear positive |  |  |
| Eriksson et al,  2001 [37] | records | questionnaire | BMI | 42% males | U - shaped in men  no association in women | insufficient data for  quantitative analysis |  |
| Frye et al,  2003 [38] | records | examination | BMI | 52% males | linear positive | insufficient data for  quantitative analysis |  |
| Fuiano et al,  2008 [39] | registry | examination | BMI | 55% males | positive * |  |  |
| Gallaher et al,  1991 [81] | records | records | weight - for - height | 50% males | linear positive |  |  |
| Gigante et al,  2003 [82] | records | examination | weight - for - height | not reported | linear positive |  |  |
| Gigante et al,  2008 [83] | examination | examination | BMI | 53% males | positive * |  | 1982: cohort identical to Barros (I) et al, 2008 |
| Gillman et al,  2003 [84] | questionnaire | questionnaire | BMI | 46% males | linear positive |  |  |
| Guimarães et al,  2006 [40] | questionnaire | examination | BMI | 50% males | positive * | birth weight dichotomized  at unjustified cut points |  |
| Hack et al,  2003 [41] | records | examination | BMI | 51% males | positive * | birth weight dichotomized  at unjustified cut points |  |
| Hawkins et al,  2008 [42] | registry | examination | BMI | 50% males | positive | Insufficient data for quantitative analysis  (birth weight as z – score) |  |
| He et al,  2000 [85] | records | records | weight - for - height | 57% males | positive * |  |  |
| Hirschler et al,  2008 [86] | interview | examination | BMI | 50% males | positive * |  |  |
| Hui et al,  2003 [87] | interview | records | weight - for - height | 49% males | linear positive |  |  |
| Hui et al,  2008 [43] | questionaire | examination | BMI | 53% males | positive | birth weight was reported as z - score |  |
| Kang et al,  2006 [88] | questionnaire | examination | BMI | 53% males | linear positive |  |  |
| Kersey et al,  2005 [89] | interview | examination | BMI | 51% males | positive * |  |  |
| Kleiser et al,  2010 [90] | questionaire | examination | BMI | not reported | positive |  |  |
| Kniażewska et al,  2006 [91] | not reported | examination | BMI | 59% males | linear positive |  | in children with hypertension |
| Koupil et al,  2008 [44] | registry | examination | BMI | 100% males | positive * | birth weight reported as mean |  |
| Kromeyer-Hausschild et al,  1999 [92] | questionnaire | examination | BMI | 49% males | linear positive |  |  |
| Laitinen et al,  2001 [93] | examination | examination | BMI | 46% males | no association |  |  |
| Leong et al,  2003 [45] | interview | interview | BMI | 100% females | U - shaped | insufficient data for  quantitative analysis | analysis only of the controls of the Collaborative Breast Cancer Study |
| Li et al,  2007 [46] | interview | examination | BMI | 54% males | linear positive | insufficient data for  quantitative analysis |  |
| Locard et al,  1992 [47] | records | examination | weight - for - height | 50% males in cases  52% males in controls | positive * | birth overweight not defined |  |
| Lundgren et al,  2003 [48] | registry | records | BMI | 100% females | linear positive | birth weight (SDS) not defined |  |
| Maddah et al,  2008 [49] | questionnaire | examination | BMI | 100% females | no association | birth weight reported as mean and s.d. |  |
| Maffeis et al,  1994 [94] | questionnaire | examination | BMI | 50% males | linear positive |  |  |
| Mangrio et al,  2010 [95] | questionaire | examination | BMI | 47,4% males | positive |  |  |
| Mardones et al,  2008 [96] | examination | examination | BMI | 51% males | linear positive |  |  |
| Mazur et al (1),  2003 [50] | questionnaire | examination | BMI | 100% females | positive * | birth weight dichotomized  at unjustified cut points |  |
| Mazur et al (2),  2003 [51] | questionnaire | examination | BMI | 100% males | positive * | birth weight dichotomized  at unjustified cut points |  |
| Mazur et al,  2008 [52] | questionnaire | examination | BMI | 51% males | positive * | birth weight dichotomized  at unjustified cut points |  |
| Meas et al,  2008 [53] | registry | examination | BMI | 43% males (SGA)  48% males (AGA) | inverse | birth weight was reported as centiles |  |
| Mikulandra et al,  2000 [97] | records | examination | BMI | 45% males | positive * |  |  |
| Miletić et al,  2004 [98] | questionnaire | examination | BMI | 100% females | linear positive |  |  |
| Mogan et al,  1986 [54] | examination | examination | weight | not reported | positive * | birth weight dichotomized  at unjustified cut points |  |
| Monteiro et al,  2003 [99] | interview | examination | BMI | 51% males | linear positive |  |  |
| Newby et al,  2005 [100] | questionnaire | interview | BMI | 100% females | U - shaped |  |  |

| O’Callaghan et al,  1997 [55] | interview | examination | BMI | 48% males | linear positive | birth weight was reported  as centile without units |  |
| --- | --- | --- | --- | --- | --- | --- | --- |
| Ochoa et al,  2007 [56] | interview | examination | BMI | 53% males | no association | birth weight dichotomized  at unjustified cut points |  |
| Oldroyd et al,  2011 [101] | interview | examination | BMI | 50,4% males | linear positive |  |  |
| Olson et al,  2008 [57] | examination | examination | BMI | 49% males | positive * | OR was reported without CI interval |  |
| Osler et al,  2008 [102] | birth certificates | examination | BMI | 100% males | positive * |  |  |
| Padez et al,  2005 [103] | questionnaire | examination | BMI | 50% males | linear positive |  |  |
| Panagiotakos et al,  2008 [104] | questionnaire | examination | BMI | 55% males | positive * |  |  |
| Péter et al,  2008 [105] | records | examination | BMI | 54% males | positive * |  |  |
| Phillips et al,  2000 [106] | records | examination | BMI | 67% males | U - shaped in men  no association in women |  |  |
| Pilpel et al,  1995 [107] | records | records | weight - for - height  skin fold thickness | 47% males | no association |  |  |
| Plagemann et al,  1997 [108] | records | examination | symmetry index | 55% males | linear positive |  | only mothers  with diabetes |
| Reilly et al,  2005 [109] | examination | examination | BMI | 51% males | linear positive |  |  |
| Rose et al,  2006 [110] | interview | examination | BMI | 51% males | positive * |  |  |
| Rugholm et al,  2005 [58] | interview | records | BMI | 51% males | linear positive | insufficient data for  quantitative analysis |  |
| Savva et al,  2005 [59] | records | examination | BMI | 53% males | linear positive | insufficient data for  quantitative analysis |  |
| Schaefer-Graf et al,  2005 [111] | records | examination | BMI | 54% males | positive * |  | only mothers with gestational diabetes |
| Seidman et al,  1991 [112] | records | records | BMI | 62% males | linear positive |  |  |
| Seidman et al,  1998 [60] | records | records | BMI | 61% males | positive * | birth weight was reported  as centile without units | only in non -  diabetic mothers |
| Serra - Majem et al,  2006 [61] | interview | examination | BMI | 50% males | positive * | insufficient data for  quantitative analysis |  |
| Shehadeh et al,  2008 [62] | records | records | BMI | 57% males | no association | insufficient data for  quantitative analysis |  |
| Sørensen et al,  1997 [113] | registry | examination | BMI | 100% males | linear positive |  |  |
| Stettler (1) et al,  2002 [114] | records | examination | BMI | 49% males | linear positive |  |  |
| Stettler (2) et al,  2002 [115] | questionnaire | examination | BMI | 50% males | linear positive |  |  |

| Stettler et al,  2003 [116] | records | examination | BMI  skin fold thickness | 54% males | linear positive |  |  |
| --- | --- | --- | --- | --- | --- | --- | --- |
| Strufaldi et al,  2008 [63] | questionaire | examination | BMI | 46% males | positive * | insufficient data for  quantitative analysis |  |
| Sugihara et al,  2008 [117] | records | examination | SDS - BMI | 47% males | no association |  | Patients with T2DM |
| Sugimori et al,  1997 [64] | questionnaire | examination | Kaup index | 65% males | positive * | birth weight dichotomized  at unjustified cut points |  |
| Takahashi et al,  1999 [65] | questionnaire | examination | BMI | 57% males | positive * | birth weight dichotomized  at unjustified cut points |  |
| Taketani et al,  1967 [118] | examination | examination | Kaup index | 65% males | positive * |  |  |
| Tene et al,  2003 [119] | questionnaire | examination | weight - for - height | 49% males | linear positive |  |  |
| Terry et al,  2007 [66] | interview | questionnaire | BMI | 100% females | linear positive |  |  |
| Tian et al,  2006 [120] | records | examination | waist circumference | 38% males | U - shaped |  |  |
| Tomé et al,  2007 [121] | records | examination | BMI | 51% males | linear positive |  |  |
| Toschke et al,  2002 [122] | questionnaire | examination | BMI | not reported | positive * |  |  |
| Turkkahraman et al,  2006 [123] | records | examination | BMI | not reported | linear positive |  |  |
| Verdy et al,  1974 [124] | interview | interview | weight | not reported | linear positive |  | only mothers  with diabetes |
| Vitolo et al,  2008 [125] | questionaire | examination | weight for height  >2 SD | 57% males | positive * |  |  |
| Vohr et al,  1980 [67] | records | examination | weight/height - Index | not reported | positive * | birth weight was reported  as centile without units | only mothers  with diabetes |
| von Kries et al,  1999 [126] | questionnaire | examination | BMI | not reported | positive * |  |  |
| von Kries et al,  2002 [127] | questionnaire | records | BMI | not reported | positive * | birth weight was reported  as centile without units |  |
| Wang et al,  2009 [128] | records | examination | BMI | 68% males | linear positive |  |  |
| Wei et al,  2007 [129] | registry | examination | BMI | 37% males | linear positive |  |  |
| Weyermann et al,  2006 [130] | interview | examination | BMI | 52% males | linear positive |  |  |
| Ylihärsilä et al,  2007 [131] | records | examination | BMI | 46% males | no association |  |  |
| Yu et al,  2008 [132] | records | examination | weight - for - height | 51% males | positive * |  |  |
| Zhang et al,  2009 [133] | records | examination | BMI | 52% males | linear positive |  |  |

* Linearity can not definitively assumed due to the fact that only two birth weight categories were used. Abbreviations: BMI, body mass index; CC, case - control - study; CO, cohort study; OR, Odds Ratio; CI, confidence interval
