# Supplementary material for: Birth Weight and Long-Term Overweight Risk: Systematic Review and a Meta-Analysis Including 643,902 Persons from 66 Studies and 26 Countries Globally
Source: PLoS One. 2012 Oct 17;7(10):e47776. doi: 10.1371/journal.pone.0047776 (PMC3474767; doi:10.1371/journal.pone.0047776)
Supplement: Table S2 — Studies that adjusted for confounders in the meta-analysis on birth weight and subsequent risk of overweight, 1966-January 2011. (DOC) [file pone.0047776.s004.doc]

**Table S2 Studies that adjusted for confounders in the meta-analysis on birth weight and subsequent risk of overweight, 1966-January 2011.**

| **Reference,**  **year** | **CONFOUNDERS ADJUSTED FOR** | | | | | | | | |
| --- | --- | --- | --- | --- | --- | --- | --- | --- | --- |
|  | **sex** | **age** | | **mat. BMI/**  **obesity** | **pat. BMI/**  **obesity** | **SES/**  **education** | **breastfed** | **ethnicity** | **other** |
| Aarup et al,  2008 [68] | **✓** |  | | **✓** | **✓** | **✓** |  | **✓** | 1. birth order |
| Armstrong et al,  2002 [70] | **✓** |  | |  |  | **✓** |  |  |  |
| Curhan et al,  1996 [77] |  | **✓** | |  |  |  |  |  |  |
| Gallaher et al,  1991 [81] | **✓** | **✓** | |  |  |  |  |  |  |
| Gigante et al,  2008 [83] |  |  | |  |  |  |  |  | 1. skin color; 2. family income |
| Hirschler et al,  2008 [86] | **✓** | **✓** | |  |  |  |  |  |  |
| Hui et al,  2003 [87] |  |  | | **✓** | **✓** |  |  |  |  |
| Kang et al,  2006 [88] |  |  | | **✓** | **✓** | **✓** |  |  | 1. TV, Computer; 2. skipping breakfast; 3. eating between meals; 4. eating fruit; 5. sleeping time |
| Kersey et al,  2005 [89] | **✓** | **✓** | | **✓** |  | **✓** | **✓** |  | 1. prematury |
| Kleiser et al,  2010 [90] | **✓** | **✓** | |  |  |  |  |  |  |
| Mangrio et al,  2010 [95] | **✓** | **✓** | | **✓** | **✓** |  |  |  | 1. maternal educational level; 2. parents’ country birth; 3. crowed living; 4. being firstborn; 5. having taken part of parental educational program and economic stress; 6. child’s intake of sweetened beverages |
| Mardones et al,  2008 [96] | **✓** |  | |  |  | **✓** |  |  | 1. BL (birth length); 2. GA (gestational age); 3. height for age at 6-8 years |
| Monteiro et al,  2003 [99] |  |  | | **✓** | **✓** | **✓** |  |  | 1. first born status |
| Newby et al,  2005 [100] |  | | **✓** | **✓** | **✓** |  |  |  | 1. smoking; 2. childhood home; 3. hormone use; 4. menopausal status; 5. Body shape at 10 years; 6. age at menarche; 7. age at first birth; 8. parity |
| Oldroyd et al,  2010 [101] |  | |  |  |  | **✓** |  | **✓** |  |
| Padez et al,  2005 [103] | **✓** | | **✓** |  |  |  |  |  |  |
| Panagiotakos et al, 2008 [104] |  | |  | **✓** | **✓** | **✓** | **✓** |  | 1. moderate to very good physical activity |
| Seidman et al,  1991 [112] |  | |  |  |  | **✓** |  | **✓** | 1. birth order; 2. maternal age; 3. area of residence at birth |
| Toschke et al,  2002 [122] |  | |  | **✓** | **✓** | **✓** | **✓** |  | 1. maternal smoking; 2. daily watching TV >1h; 3. sports outside school; 4. having siblings |
| Wang et al,  2009 [128] |  | |  |  |  | **✓** |  |  | 1.mother’s age; 2.mother’s body mass index before pregnancy  3. feed method after birth; 4. current outdoor activity time in hour per day; 5. current TV in hour per day |
| Zhang et al,  2009 [133] | **✓** | | **✓** |  |  | **✓** |  |  | 1.area; 2.gestational age; 3.history of illness status; 4.current health status; 5. health status of mother/father. |
