# Supplementary material for: Birth Weight and Long-Term Overweight Risk: Systematic Review and a Meta-Analysis Including 643,902 Persons from 66 Studies and 26 Countries Globally
Source: PLoS One. 2012 Oct 17;7(10):e47776. doi: 10.1371/journal.pone.0047776 (PMC3474767; doi:10.1371/journal.pone.0047776)
Supplement: Table S3 — Studies that reported data on neonatal weight gain or infant growth in the meta-analysis on birth weight and subsequent risk of overweight, 1966-January 2011. (DOC) [file pone.0047776.s005.doc]

**Table S3 Studies that reported data on neonatal weight gain or infant growth in the meta-analysis on birth weight and subsequent risk of overweight, 1966-January 2011.**

| **Reference** | **Definition of rapid weight gain** | **Effect of birth weight on overweight**  **unadjusted adjusted for weight gain**  **OR (95% CI) OR (95% CI)** | | **Effect of weight gain on overweight**  **unadjusted adjusted for birth weight**  **OR (95% CI) OR (95% CI)** | |
| --- | --- | --- | --- | --- | --- |
| Reilly et al,  2005 [109] | Weight at 12 months - birth weight | 1.05 (1.03-1.07)  (per 100g increase) | -^§^ | 1.07 (1.05-1.10)  (per 100g increase) | 1.06 (1.02-1.10) |
| Stettler (1) et al,  2002 [114] | Weight at 12 months - birth weight | 1.47 (1.23-1.76)  (per kg increase) | -^§^ | 1.46 (1.33-1.60)  (per kg increase) | 1.46 (1.27-1.67) |
| Dennison et al,  2006 [79] | Weight at 6-months - birth weight | 1.05 (1.02-1.09)  (per 100g increase) | 1.07 (1.04-1.11) | 1.37 (1.23-1.53)  (in 100g per mo) | 1.43 (1.27-1.60) |
| Stettler (2) et al,  2002 [115] | Weight at 4 months - birth weight | 1.07 (1.05-1.08)  (per 100g increase) | 1.06 (1.04-1.08) | 1.29 (1.25-1.33)  (in 100g per mo) | 1.38 (1.32-1.44) |
| Stettler et al,  2003 [116] | z score from birth to 4 months (1 SD) | 1.50 (0.56-4.01)  (per kg increase) | 5.69 (0.73-44.0) | 3.00 (1.33-6.76)  (> 1 SD increase) | 6.72 (1.93-23.4) |

^§^ Reilly et al, 2005 and Stettler (1) et al, 2002 reported an adjusted model containing birth weight, but OR is not shown for this variable.
